# Exploring Professionalism among Final Year Dental Students and New Graduates: Translating Knowledge to Practice

**DOI:** 10.1055/s-0042-1743153

**Published:** 2022-06-07

**Authors:** Khaled Khalaf, Mohamed El-Kishawi, Sausan Al Kawas

**Affiliations:** 1 College of Dental Medicine, University of Sharjah, Sharjah, UAE; 2 Institute of Dentistry, University of Aberdeen, Aberdeen, United Kingdom

**Keywords:** professionalism, dental graduate, knowledge, practice, application, competency

## Abstract

**Objectives** The aim of this study was to evaluate senior students' and dental graduates' perception of professionalism as defined by international regulatory bodies and to assess students' ability to apply such knowledge in clinical-based scenarios.

**Materials and Methods** Knowledge of professional competencies was conducted using a survey based on domains of professionalism for a newly qualified general dental practitioner as defined by international regulatory bodies. This survey consists of 32 items addressing participants' perception of three domains of professionalism. Application of the perceived professional competencies was tested by asking participants to answer questions following the observation of a clinical-based scenario video where participants needed to identify issues related to professional, ethical, and communication skills, which were embedded in the scenarios.

**Statistical Analysis** Differences were analyzed using ANOVA and t-tests ( *p* <0.05).

**Results** Data from 146 participants showed no significant differences in the survey scores between the participants' professional levels, place of practice, age groups, and genders. Analyses of correlation between the different domains of professionalism showed significant relationships between pair-wise comparisons of the total domain scores. Paired *t* -tests revealed that the mean score percentage for each of the three domains of professionalism was significantly higher than the responses reported in the relevant clinical-based scenario questions.

**Conclusions** This indicates that all domains of professionalism contributed in a similar way to their overall assessment. Furthermore, our findings show that dental practitioners may not be able to apply their knowledge of professionalism in real-life scenarios. This suggests that teaching professionalism at the early stages of the dental curriculum should incorporate innovative approaches to mimic real-life scenarios.

## Introduction

Although it is difficult to have a concrete definition of professionalism as a competency, many have identified common domains that underpins this term in health care provision. 1 2 3 Specifically in dentistry, several organizations and regulatory bodies worldwide stated that dental graduates are required to have minimum standards of clinical skills as well as soft skills, i.e., professionalism and communication skills to adequately perform various procedures during their routine daily dental practice. 4 5 6 Furthermore, there has been more emphasis in recent years on professionalism as an important attribute to the skills set of a general dental practitioner. 4 6 Clinical skills have long been well taught and assessed in dental curricula, 7 but there is no consensus as yet on the teaching and assessment of the soft skills, especially professionalism in both undergraduate and postgraduate clinical curricula. 8 9 10 11

This is due to the multifaceted nature of this skill, especially when interpreted in different cultures, contexts, and secular changes in the society. 12 13 14 15 16

Therefore, it is important to develop teaching methods and assessment of professionalism in the contemporary dental curriculum to meet the increased demand of the society and regulatory bodies, with clear and objective learning outcomes in parallel to teaching and assessment of the diagnostic and manual dexterity skills of clinical procedures. 17 18 19

At the University of Sharjah, we have an integrated, competency-based 5-year Bachelor of Dental Surgery (BDS) curriculum. It is a theme-based curriculum with four main themes, that is, Human Biology, Community Dentistry, Dental Health Sciences, and Dental Clinical Practice, with the first two themes ceasing at the second and third years, respectively, whereas the other two themes span over the whole curriculum. Ethics and professionalism are mainly taught and assessed as part of the Dental Clinical Practice theme in the final year of the BDS program, using conventional teaching methods and assessments, that is, lectures and written continuous and end-of-year examinations. 20 In the view of the increasing importance of professionalism as an essential part of the skills set of a newly graduated general dental practitioner and the need to have reciprocity in the teaching and assessment of this skill, we conducted this study with the aim to evaluate our senior students' and dental graduates' perception of professionalism as defined by international regulatory bodies and to introduce a clinical-based scenario video to assess whether our students could apply their prior knowledge of professionalism in their daily clinical practice.

## Materials and Methods

### Participants

An ethical approval was granted from the University of Sharjah prior to conducting the study (ethical approval number: REC-19-12-10-01). The main investigator introduced the research project through an e-mail invitation to final-year students at College of Dental Medicine, University of Sharjah; internship students at University of Sharjah; and graduates of the University of Sharjah who are either currently enrolled in a master's degree program or have been working as General Dental Practitioners (GDPs) for 3 years following graduation. All participants were provided with information sheets and consent forms to voluntarily take part in the study. As a result, a total of 146 participants (33 final year BDS students, 30 interns, 9 graduate students, and 74 GDPs) consented to participate in the study.

### Professional Competency Knowledge Survey

Knowledge of professional competencies was conducted using a survey based on domains of professionalism deemed to be necessary for a newly qualified general dental practitioner in the United States, Australia, Canada, Europe, and Pakistan. 4 21 22

This survey (Appendix 1) consists of 32 items that are rated on a 5-point Likert's scale: 1 (strongly disagree), 2 (disagree), 3 (not sure), 4 (agree), and 5 (strongly agree). These items address participants' perception of three domains of professionalism under which were grouped 32 competencies as follows: 9 for Professional Attitude and Behavior (PAB), 15 for Ethics and Jurisprudence (EJ), and 8 for Communication and Interpersonal Skills (CIS). A pilot study was performed by delivering the questionnaire to 25 randomly selected dental graduates to test the clarity, comprehension and the feasibility of completing the questionnaire in a reasonable time frame. Subsequently, it was found that all items of the questionnaire were clear and meaningful to participants following a minor revision being made to some items. Moreover, it was found that participants require, on average, 13 minutes to complete the questionnaire. Furthermore, the survey has shown good internal reliability (Cronbach's α of 0.87). Data on participants' personal characteristics and demographic data, that is, age, gender, year of graduation, professional level, and type of institution/employer were also collected. An overall score for each individual was calculated by assigning a value of 1 for “strongly disagree,” 2 for “disagree,” 3 for “not sure,” 4 for “agree,” and 5 for “strongly agree”; and percentages of these scores were then summed.

### Application of Professional Competency Knowledge/Clinical-based Scenario Questions

Application of the perceived professional competencies was tested by creating a clinical-based scenario video (Appendix 2), where dental practitioners needed to apply previously learned professional, ethical, and communication skills. We asked participants who completed the knowledge survey to answer five questions related to the video, of which two were related to professional attitude and behavior (question 3, which is called application 1 of the domain, and question 4, which is called application 2 of the domain), two questions were related to ethics and jurisprudence (question 1, which is called application 1 of the domain, and question 2, which is called application 2 of the domain), and one question related to communication and interpersonal skills (question 5, which is called application 1 of the domain). The five questions were single best answer questions, but with a hierarchy of level of plausibility ( Table 1 ). They were written by two experts in dental education and further reviewed by a panel of clinical academics who had interest and significant experience in dental education. An overall score for each participant was calculated by assigning a value from 1 to 5 from the least to the most accurate/plausible response, and percentages of these scores were then summed.

**Table 1:** Mean score percentages and (standard deviation) of the correct answers to the five questions and the percentages of participants who answered each option related to the clinical-based scenario video

	Questions and answers (scores)	%	*M* (SD)
A)	Professional Attitude and Behavior		
Q3.	Displaying appropriate caring behavior toward patients and showing willingness to help		81.32 (23.88)
Application 1	a. Dr. Amel was emotional in her conversation with Dr. Shareef (2)	35.6	
	b. Dr. Shareef had an insight into his behavior, but thought the matter was trivial (4)	60.9	
	c. Dr. Shareef admitted his mistake and showed remorse (1)	0	
	d. Dr. Shareef's behavior can lead to disciplinary action (3)	3.5	
Q4.	Displaying professional behavior toward all members of the dental team		54.02 (29.50)
Application 2	a. It is determined that this incident was an isolated one (4)	16.1	
	b. Dr. Shareef appreciates the significance of his actions (3)	29.9	
	c. Dr. Shareef takes steps to change his behavior (2)	8.0	
	d. Dr. Amal has concern that Dr. Shareef may raise the matter to her line manager (1)	46.0	
B)	Ethics and Jurisprudence		
Q1.	How should Dr. Ahmed respond to the question: “Do you have a problem with that?		87.05 (18.95)
Application 1	a. Say “no” and walk away (1)	4.5	
	b. Apologize and walk away (2)	1.2	
	c. Tell Dr. Shareef the patient's story (3)	2.3	
	d. Say something about the rules regarding patient confidentiality (5)	53.4	
	e. Explore other options around how to discuss the rules and regulations of patient confidentiality with a colleague or chief of staff (4)	38.6	
Q2.	Is Dr. Shareef's response appropriate from an ethical point of view?		51.14 (7.49)
Application 2	a. Yes (1)	97.7	
	b. No, the reason he gave for accessing the chart in the first place (2)	2.3	
C)	Communication and Interpersonal Skills		
Q5.	Establishing a patient–dentist relationship that allows the effective delivery of dental treatment including, when appropriate, a relationship with a parent or carer		35.06 (12.33)
Application 1	a. This action generalizes the problem and allows others to benefit (4)	0	
	b. This course of action might help Dr. Shareef to view the conversation in a more positive light (3)	0	
	c. Dr. Amal decided to let people know that Dr. Shareef has such a behavior (1)	59.8	
	d. This action will serve as an apology from Dr. Shareef to Dr. Ahmed in public (2)	40.2	

### Data Analysis

All data were coded prior to data analysis. Tests of normality were performed where appropriate using normal probability plots 23 and equal variance tests. 24 Percentages of responses and the overall score for each individual were calculated. Mean values and standard deviations of the surveys were calculated according to the method described by Field. 25 Internal consistency reliability was assessed by Cronbach's α (α). 26 Pearson's product–moment correlations were used to test the association between scores of the surveys. Analysis of variance (ANOVA), with post hoc analyses and student's *t* -test as appropriate, was used to assess the effect of differences in gender, professional level, place of practice, and age groups on the outcome survey scores within the three domains of the knowledge survey. Student's *t* -tests were used to assess whether there were statistically significant differences in score percentages between each of the perceived domain of professionalism and the responses to the clinical scenario video questions relevant to that domain. All data analyses were conducted using SPSS (version 26.0; SPSS Inc, Chicago, Illinois, Unites States), and statistical significance for quantitative and categorical data was set at *p* < 0 0.05. Responses from the open-ended questions were analyzed using a thematic analysis method as described by Braun and Clarke. 27

## Results

Data from 146 participants (41 males and 105 females) were available for analyses. Participation response rate was 75%. Participants' ages ranged from 22 to 33 years (mean age and standard deviation: 25.64 ± 2.43 years). Participants' professional level varied between final-year dental students (23%), internship dental graduates (20%), postgraduate students (6%), and general dental practitioners (51%) graduated from the University of Sharjah ( Table 2 ). The majority of participants were Arabs (90%), and the remaining minority (10%) were either Persian, Indian, or Pakistani.

**Table 2:** Survey scores (percentages) of the three professional domains (PAB, EJ, and CIS), means and (standard deviations) of participants by gender, professional level, place of practice, and age group

	*N*	PAB	EJ	CIS
		*M* (SD)	*M* (SD)	*M* (SD)
Gender				
Male	41	89.60 (8.51)	90.80 (8.41)	89.51 (9.05)
Female	105	90.29 (9.34)	89.29 (10.95)	87.79 (12.82)
Professional level				
BDS5	33	90.91 (8.74)	91.07 (9.68)	89.85 (9.29)
Intern	30	88.74 (9.78)	85.73 (12.49)	85.83 (13.51)
Postgrad	9	88.89 (11.17)	92.73 (8.78)	89.44 (10.95)
GP	74	90.42 (8.82)	90.36 (9.49)	88.41 (12.38)
Place of practice				
UDHS	75	89.51 (9.14)	88.44 (10.78)	87.60 (11.30)
Private practice	49	90.43 (8.59)	90.40 (9.04)	88.52 (10.58)
UDHS and private	7	93.34 (8.60)	94.47 (6.50)	95.00 (5.20)
Unemployed	5	91.12 (8.48)	93.34 (8.75)	90.00 (10.75)
Academic	4	92.78 (8.78)	95.65 (5.28)	91.25 (11.81)
Government	6	88.13 (15.13)	87.55 (18.25)	83.33 (28.45)
Age group (y)				
< 25	53	91.78 (8.84)	91.37 (9.24)	89.86 (9.97)
25–26	50	89.34 (8.95)	88.93 (11.45)	87.80 (12.31)
> 26	43	88.89 (9.46)	88.58 (10.09)	86.86 (13.50)

Abbreviations: CIS, Communication and Interpersonal Skills; EJ, Ethics and Jurisprudence; PAB, Professional Attitude and Behavior; UDHS, University Dental Hospital Sharjah.

### Responses to the Knowledge Survey

The professionalism competency survey had an excellent internal reliability (Cronbach's α of 0.97). Responses to individual items within the scale ranged from 0 to 80% ( Table 3 ). It was clear that the majority of participants responded with the “strongly agree” and “agree” categories within the three domains in this survey. ANOVA analysis showed no statistically significant differences in survey scores between professional levels, place of practice, and age groups ( Table 2 ). Similarly, no statistically significant differences in the survey scores were found between genders. Analyses of correlation between the different domains of professionalism showed statistically significant relationships between the total domain scores for the PAB and EJ ( *r* (146) = 0.83, *p* < 0.001), the PAB and CIS ( *r* (146) = 0.81, *p* < 0.001), and the EJ and CIS ( *r* (146) = 0.86, *p* < 0.001).

**Table 3:** Participants' perception on professional competencies in dentistry

	Questions	%SA	%A	%N	%D	%SD
A)	Professional Attitude and Behavior					
1)	Displaying appropriate caring behavior toward patients and showing willingness to help	80	19	1	0	0
2)	Displaying professional behavior toward all members of the dental team	77	22	1	0	0
3)	Have knowledge of the management of a dental practice	55	36	8	1	0
4)	Deal with other members of the dental team with regard to health and safety	64	32	4	0	0
5)	Have knowledge of the importance of health in relation to occupational hazards and its impact	58	36	6	0	0
6)	Have knowledge of the social and psychological issues relevant to the care of patients	51	40	8	1	0
7)	The management of a dental practice, patient communication, and able to oversee financial aspects of practice	49	33	14	3	1
8)	Managing and maintaining a safe working environment	65	33	2	0	0
9)	Seeking continuing professional development (CPD) on an annual basis, demonstrated through portfolio/CPD Logbook	40	42	15	3	0
B)	Ethics and Jurisprudence					
10)	Providing humane and compassionate care to all patients	70	26	3	1	0
11)	Practicing with personal and professional integrity, honesty, and trustworthiness	71	26	2	1	0
12)	Respecting patients and colleagues without prejudice concerning gender, diversity of background opportunity, language, culture, disabilities, and sexual orientation	70	24	4	1	1
13)	Recognizing their own limitations	58	38	3	1	0
14)	Producing and maintaining an accurate patient record and record of patient treatment	66	30	3	1	0
15)	Selecting and prioritizing treatment options that are sensitive to each patient	62	33	5	0	0
16)	Recognizing patients' rights, particularly with regard to confidentiality, informed consent, and patients' obligations	69	27	3	1	0
17)	Have knowledge of the ethical principles relevant to dentistry	60	36	3	1	0
18)	Acknowledging that the patient is the center of care and that all interactions, including diagnosis, treatment planning, and treatment, must focus on the patient's best interests	66	30	3	1	0
19)	Have knowledge of the fact that dentists should strive to provide the highest possible quality of patient care in a variety of circumstances	60	36	3	1	0
20)	Taking action that is audit and clinical governance to ensure quality of care	45	43	10	1	1
21)	Selecting and prioritizing patient's individual needs, goals, and values, compatible with contemporary methods of treatment, and congruent with an appropriate oral health care philosophy	52	40	6	1	1
22)	Have knowledge of the socioeconomic inequities and inequalities in oral health	42	45	11	2	0
23)	Taking appropriate action to help the incompetent, impaired, or unethical colleague and their patients	43	40	14	2	1
24)	Have knowledge of the judicial, legislative, and administrative processes and policy that impact all aspects of dentistry	41	34	21	3	1
C)	Communication and Interpersonal Skills					
25)	Establishing a patient–dentist relationship that allows the effective delivery of dental treatment including, when appropriate, a relationship with a parent or carer	61	36	2	1	0
26)	Identifying patient expectations, desires, and attitudes (needs and demands) when considering treatment planning and during treatment	60	38	1	1	0
27)	Sharing information and professional knowledge with the patient	60	36	3	1	0
28)	Identifying the psychological and social factors that initiate and/or perpetuate dental, oral, and facial disease and dysfunction and diagnose, treat, or refer, as appropriate	53	38	8	1	0
29)	Have knowledge of the behavioral sciences including behavioral factors (including factors such as ethnicity and gender) that facilitate the delivery of dental care	48	40	9	2	1
30)	Have knowledge of the role and the stages of the intellectual, social-emotional, and language development of children and adolescence	45	45	9	1	0
31)	Applying principals of stress management to oneself, to patients, and to the dental team as appropriate	50	38	9	3	0
32)	Communicating with other doctors and health professionals, verbally and in writing including being able to receive and give constructive criticism	46	40	9	4	1

Abbreviations: %A, percentage of agree response; %D, percentage of disagree response; %N, percentage of neutral response; %SA, percentage of strongly agree response; %SD, percentage of strongly disagree response.

### Responses to the Application of Knowledge Questions

**Fig. 1:**
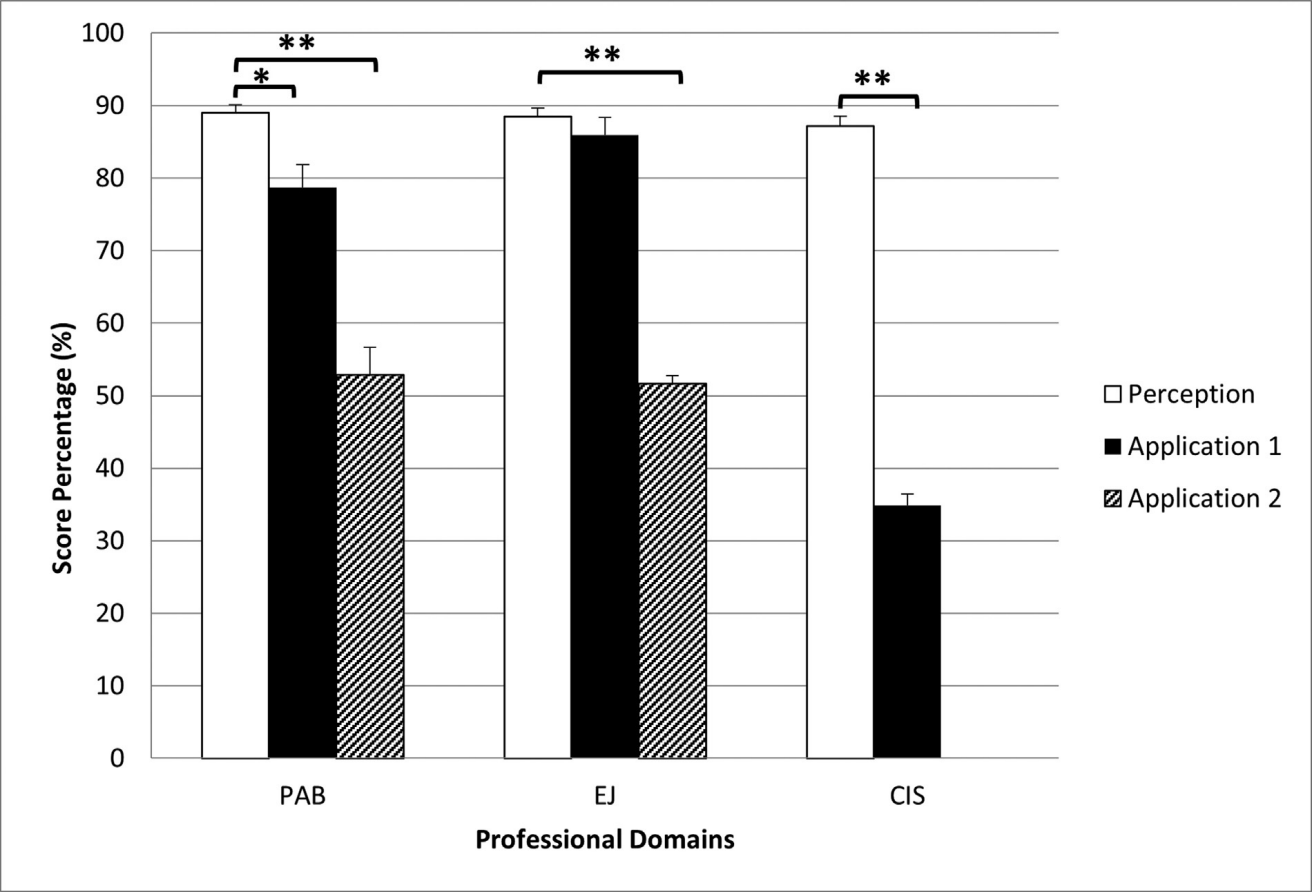
Differences between the mean survey scores (perception) of the three professional domains (PAB, EJ, and CIS) and their relevant response scores (applications 1 and 2) to the clinical video scenarios. PAB, Professional Attitude and Behavior; EJ, Ethics and Jurisprudence; and CIS, Communication and Interpersonal Skills; applications 1 and 2, relevant questions to the domains; error bars= +1 standard error. Significant difference between survey scores, error bars = +1 standard error. * *p* < 0.05; ** *p* < 0.01.

Mean score percentages of the correct answers to the five questions ranged from 35.1 to 87.1. The percentages of participants who answered each option related to the clinical-based scenario video ranged from 1.1 to 97.7% ( Table 1 ). Paired *t* -tests were used to assess whether there were statistically significant differences in the mean knowledge/perception survey scores of the three domains of professionalism (PAB, EJ, and CIS) and the response scores to their relevant clinical scenario video questions (applications 1 and 2; Fig. 1 ). Paired *t* -tests revealed that the mean score percentage for the PAB domain was significantly higher than that of question 3 ( *t* (60) = 3.16, *p* = 0.002) and question 4 ( *t* (60) = 9.22, *p* < 0.001). The mean score percentage for the responses reported for question 2 was significantly lower than the mean score perception reported in the EJ domain ( *t* (60) = 21.32, *p* < 0.001). Similarly, the mean score percentage for the responses reported for question 5 was significantly lower than the CIS domain mean score ( *t* (60) = 25.95, *p* < 0.001).

### Thematic Analysis of Participants' Responses to the Open-ended Questions

Seventy-eight of 88 participants (89%) responded to the question explain your answer to “how should the clinician responsible for patient's care respond to the question: ‘Do you have a problem with that?’” Most participants explained that the supervisor in the scenario was breaching patient's confidentiality by checking the patient's personal details and dental records. The second reported explanation was unprofessional behavior and the aggressive attitude by the supervisor toward the intern. It was also mentioned that the supervisor must have proper consent and permission from the patient to access their details ( Table 4 ).

**Table 4:** Thematic analysis of participants' response to the open-ended question

#	Themes	Subthemes	Quotations
1	Patient's confidentiality	• Crossing patient confidentiality • Using patient details for personal reason • Violating rules and not respecting confidentiality	“He is crossing the patient confidentiality in two ways: He is checking a patient dental record who is not registered under his name and is discussing the reason for that with others” “Dr. Shareef is using patients' own details that are very confidential in the hospital for his own personal life; in addition to that, he is sharing the patient's personal info with another colleague and that is unethical” “The Dr. is violating the rules and regulations and patient confidentiality, which is unethical” “He must respect patient's confidentiality”
2	Professional attitude and behavior	• Not appropriate behavior in the clinic • Lack of respect for intern • Not suitable behavior	“The language (as well as the body language he expressed) and his tone are not suitable and professional for a clinic setting” “he shouldn't respond to the intern with an aggressive tone and he should show mutual respect” “He should be more polite and have better attitude” “Aggressive and disrespectful”
3	Patient's consent	• Not taking permission from institute or colleagues • No consent from patient • Violation of rules and regulations	“Dr. Shareef should take permission from the doctor who is treating that patient” “He does not have a proper appointment date and wasn't assigned for that patient” “IF the patient is not your patient and you want to read their file, you must have at the very least the consent of said patient”

## Discussion

The majority of participants responded with the “strongly agree” and “agree” categories within the three domains of the professionalism perception competency survey, indicating that the participants perceived all items of professionalism to be important, regardless of their professional levels, place of practice, age, and gender. In our study, the ranges of participants' responses to the three domains of professionalism survey, that is, “professional attitude and behavior: PAB,” “ethics and jurisprudence: EJ,” and “communication and interpersonal skills: CIS” were 82 to 99%, 75 to 98%, and 86 to 98%, respectively. These were higher than what was reported in a previous investigation of perception of professionalism among 386 dental graduates of eight public- and private-sector dental colleges in Pakistan, 22 where the corresponding figures to those in our study were 51 to 99%, 60 to 97%, and 61 to 91%. The differences between our findings and those reported in the aforementioned study may be attributed to the differences in teaching and application of the professionalism subject in the BDS curricula. Further support to this explanation can be inferred from previous investigations that showed inadequate infection control practices among senior faculty members in Karachi, Pakistan, 28 as compared with their prior knowledge of such measures, and the high prevalence of musculoskeletal disorders among general dental practitioners in Pakistan, which is most probably due to poor teaching and application of proper ergonomics during their BDS program. 29 Furthermore, many dental curricula in developing countries may not prepare their students to work well in disadvantaged populations with special oral health needs. 30 It is part of our dental curriculum to make our students fully aware of the factors that have an impact on the oral health needs of the community including socioeconomic inequities. Furthermore, we have a mobile dental clinic with regular students' allocation to serve the rural, elderly, and disadvantaged populations.

In our study, participants did not consider maintaining Continuing Professional Development (CPD) as important as the other items of the PAB domain. This is due to the need to invest a lot of time and money in activities that may not be considered important, especially those that were deemed noncompulsory by regulatory bodies. 22 31 The item that was considered by our participants as the least important among all domains of professionalism was “have knowledge of the judicial, legislative and administrative processes and policy that impact all aspects of dentistry.” A similar finding was also reported in previous studies, 22 32 which may be attributed to the lack of a formally structured and taught course addressing ethics and legal issues in many dental curricula. 33

Looking closely at the responses to the items within each domain, in the “PAB” domain, items that were considered most important by participants were “displaying appropriate caring behaviour towards patients and showing willingness to help” and “displaying professional behaviour towards all members of the dental team,” whereas the item that was deemed the least important was “seeking continuing professional development (CPD) on an annual basis, demonstrated through portfolio/CPD Logbook.” In the “EJ” domain, participants considered “providing humane and compassionate care to all patients” and “practicing with personal and professional integrity, honesty and trustworthiness” as the most important items, whereas the item “have knowledge of the judicial, legislative and administrative processes and policy that impact all aspects of dentistry” was considered the least important. In the “CIS” domain, the items “establishing a patient-dentist relationship that allows the effective delivery of dental treatment including, when appropriate a relationship with a parent or carer” and “identifying patient expectations, desires and attitudes (needs and demands) when considering treatment planning and during treatment” were deemed by participants as the most important ones, whereas the item “communicating with other doctors and health professionals, verbally and in writing including being able to receive and give constructive criticism” was considered the least important item. The above findings agree with the study by Choudhry et al 22 where a similar pattern of importance was reported by their participants.

The correlation between the different domains of professionalism showed significant relationships between the total domain scores for the PAB and EJ, the PAB and CIS, and the EJ and CIS ( *p* < 0.001). This indicates that all domains of professionalism are interrelated and if participants have a good knowledge of one domain, they are very likely to be good at the remaining domains of professionalism. Furthermore, this provides an evidence to the appropriate use of the questionnaire as the three domains contributed in a similar way to the overall assessment of professionalism.

To investigate the relationship between knowledge and practice of professionalism, a correlation was tested between the mean of the three professional domains (PAB, EJ, and CIS) and their relevant response scores (applications 1 and 2). It was found that the mean score percentage for the PAB domain was significantly higher than the responses reported in questions 3 and 4, which were meant to test the application of the knowledge in the PAB domain. This means that participants' knowledge of the PAB domain was significantly better than their application of such knowledge when tested in clinical practice. Similarly, participants' knowledge of the EJ and CIS domains were significantly better than their application of such knowledge. These differences between the knowledge of the three domains of professionalism and their application in clinical practice suggest that teaching professionalism should start with a cognitive base, reinforced through case-based scenarios, and internalized throughout the clinical practice phase of the curriculum (experiential learning). 34 This necessitates a strong support of the teaching of professionalism throughout the educational program.

## Conclusion

- All aspects of professionalism are interrelated and the three domains contributed in a similar way to the overall assessment of professionalism.
- Dental practitioners may not be able to apply their knowledge of professionalism in real-life scenarios.
- Teaching professionalism at the early stages of the curriculum should incorporate innovative approaches to mimic real-life scenarios and should be reinforced throughout clinical practice at the later stages of the curriculum.
